# Impact of weather and climate advisories on agricultural outcomes in Pakistan

**DOI:** 10.1038/s41598-023-51066-4

**Published:** 2024-01-10

**Authors:** Mani Nepal, Muhammad Ashfaq, Bibek Raj Sharma, Mandira Singh Shrestha, Vijay Ratan Khadgi, Marta Bruno Soares

**Affiliations:** 1 South Asian Network for Development and Environmental Economics (SANDEE), GPO Box 3226, Kathmandu, Nepal; 2 International Center for Integrated Mountain Development (ICIMOD), Lalitpur, Nepal; 3 Muhamad Nawaz Shareef University of Agriculture, Multan, Pakistan; 4 University of Leeds, Leeds, UK; 5 Met Office, Leeds, UK

**Keywords:** Climate sciences, Environmental sciences, Environmental social sciences

## Abstract

The earth is warming, and the frequency of extreme weather events have been rapidly growing globally with unprecedented consequences to farming communities. In principle, weather and climate information services (WCIS) can help farmers better manage their activities and farm level outcomes by supporting their decision-making with relevant and usable information to address the potential impacts of expected changing climate conditions. But, in practice, can the availability and use of WCIS help improve agricultural outcomes given the weather and climate related uncertainties? To understand the use and impact of WCIS in the cotton-wheat cropping areas of Pakistan, we conducted a multistage stratified clustered random sample of 612 farm households in the provinces of Punjab and Sindh. Over 55% of the farm households in the sample indicated that they used WCIS provided by the Pakistan Meteorological Department and other sources for making their agricultural decisions. Our analysis, however, suggests that the impact of using WCIS on major farm outcomes (i.e. farm revenue, costs, profits, and input usage) is not statistically significant when compared with those farm households not using WCIS (null result). This result is robust to different estimation techniques (i.e. ordinary least squares, instrumental variable approach, and propensity score matching method). From the focus group discussions, we gathered that farmers perceived WCIS as less reliable, often unclear, and difficult to understand as this information is not translated and transmitted in local languages. Addressing these issues can help reduce the impact of climate and weather variability on farm outcomes in those provinces as well as in Pakistan more generally. Our study suggests that, under uncertainty, emphasis should be on WCIS that farmers can rely on for making farming related decisions.

## Introduction

The earth is warming resulting in changing climate conditions such as an increase in extreme events e.g. high-intensity short-duration rainfall events, frequent floods, droughts, and heat waves which are affecting the lives and livelihoods of people globally^1^. Low and middle-income countries tend to be more vulnerable to climate change compared to high-income countries^2^. Nevertheless, there is a variation in the rise in temperature in different countries. For example, whilst the increases in annual mean temperature in China in the past 115 years (1901–2015) was 1.12 °C with 0.10 °C per decade^3^, in Pakistan, the mean annual raise in temperature was much higher, with around 0.36 °C per decade over the past fifty years (1952–2009)^4^. In Pakistan, the decade of the 2000s was the warmest compared to earlier decades^5^, suggesting a differential degree of climate change in different countries and different time periods.

Studies suggest that an increase in temperature and extreme events affect crop yield^6,7^. In South Asia, it is estimated that rainfed maize yield will decline by 12% and irrigated maize will decline by 14% by 2050 given expected changes in climate^8^. Literature suggests that around 50% of yield reduction in agronomic crops can be attributed to extreme weather events globally^5^. As a result of climate change, around 23% of land area and 50% of the population in Pakistan are expected to be severely affected in future^9^.

In principle, the use of weather and climate information services (WCIS) can help farming communities to make better and more informed decisions that help mitigate the impacts of weather and climatic variation and yield socio-economic benefits^10–13^. Weather and climate forecasts and their timely dissemination to relevant users can help increase agriculture production, reduce losses, and increase efficiency regarding key inputs in farming such as water for irrigation, energy, and labour^14^. Ray et al.^15^ suggests that farmers in Odisha in India who subscribed and adopted agro-met advisory services (AAS) gained an additional benefit of 41.2%, 20.8%, and 34.8%, in green gram, rice, and maize crops, respectively. An assessment from Uttar Pradesh in India, showed that farmers who adopted AAS in real-time obtained a 22% higher net return in wheat crops^16^.

Pakistan, an agriculture-dependent developing country with a 22% contribution of the agricultural sector to the national economy, is among the most vulnerable nations due to the impacts of climate extremes owing to its limited adaptive capacity^17^. The country is ranked fifth among the most vulnerable nations from a climate change perspective^18^, where the effect has been reflected in terms of a rise in sea level, glacier retreat, intensive and more frequent floods as well as droughts^19,20^.

The agriculture sector, which provides employment to 37.4% of the labor force in Pakistan, has grown at a rate of 4.4% between 2021 and 2022^21^. In addition, the rapid growth of the population, unplanned urbanization, and heavy dependency on natural resources put pressure on the environment which in turn may amplify the impacts of climate change^22^. Against this backdrop, comprehensive planning and actions to reduce the economic and ecological impacts of extreme weather events and changing climate are required to feed a fast-growing population. In this context, WCIS can support farmers in better managing current and future risks^23^. In their planning and day-to-day activities, farmers need to manage weather and climate variability, which involves a high degree of uncertainty and affects crop planning at every level, from variety selection, the sowing/planting to harvesting and the final stage of processing and storage of the produce^24^. Current knowledge and evidence of the effectiveness and impact of using WCIS is limited and disparate. For example, Mahato et al.^24^ suggest that the use of on-time weather-related reliable advisories may help farmers for reducing inputs cost, increase farm revenue, and also increase farm profit since such information helps farmers to make the right decision on time. If the farmers receive reliable weather forecasts in advance and act upon such information in a timely manner, crop loss could be minimized^25^. On the other hand, Sharma et al.^26^ reports that farmers may not change their agricultural practices even if they receive WCIS. The literature on the impact of using WCIS is also limited and results depend on the context, country and methods used for analysis^27^.

Given such a thin but contrasting evidence, our study aimed to examine the effect of using WCIS on specific outcomes such as farm income, cultivation cost, and farm profits using the cotton-wheat growers in Pakistan as a case study. More specifically, our research questions is: can the availability and use of weather and climate information services (WCIS) help improve agricultural outcomes in Pakistan given the weather and climatic uncertainties? We also examine the effect of WCIS on input costs such as the cost of irrigation, fertilizer, and agrochemicals (the three most important agricultural inputs with wider environmental implications). For example, excessive use of agrochemicals, such as pesticides, not only increases cultivation costs but also harms human as well as environmental health and destroys natural pollinators such as bees and other insects and microorganisms which eventually reduces biodiversity and crop yields^28^. We hypothesize that under uncertain variation in weather and climatic factors, the use of appropriate WCIS can help farming communities making better decisions regarding the choice, timing, and use of the farm inputs so that it helps reducing the costs and increasing the farm revenue and profits. With this analysis our study also aims to contribute to existing literature on the potential impacts of using WCIS on farm outcomes.

Using a multi-stage stratified clustered random sample of 612 farm households following a wheat–cotton cropping pattern (rotating cotton after wheat, which are two of the most dominant crops in Pakistan) in the Punjab and Sindh provinces of Pakistan, we estimated different econometric models using three different types of estimation techniques—ordinary least squares, instrumental variable approach, and propensity score matching. In the sample, over 55% of the farm households were using WCIS. Our results suggest that farmers who currently use WCIS did not benefit from an increase in profit or revenues or incurred lower costs by saving on key farm inputs while cultivating wheat–cotton crops. These null results are robust to alternative estimation techniques and model specifications, suggesting that the existing system of providing WCIS in the country needs to be reexamined and enhanced so that farmers receive and use the relevant WCIS as and when they need to make farming decisions under uncertain weather and climate conditions.

## Weather and climate services in Pakistan

The Pakistan Meteorological Department (PMD) is the mandated organization to generate and distribute WCIS in the country. PMD is responsible for providing meteorological services across the country to a wide variety of users, who require weather and climatic information^29^. Since 1988, agro-met services have been in operation with a National Agro-met Centre and five regional centres. PMD currently produces agro-met advisories for the farming communities which includes the weekly weather forecast along with crop-specific suggestions. In addition, PMD also prepares and disseminates monthly, seasonal, and crop-specific forecasts. PMD disseminates its WCIS using a range of mechanisms including its website, SMS, television, radio broadcasts, and newsletters. Other PMD products include a three-day agro-met forecast daily disseminated on its website, a weekly agro-met forecast every Monday in a newsletter, and agro-met bulletins with analysis on a weekly, 10 days, and monthly basis using social media. In its communications, PMD uses national as well as some of the major local languages.

Over 55% of the sampled farm households used WCIS during 2021 (the year when the survey data was collected). Weather news on national TV and SMS were the main mechanisms for receiving WCIS for nearly 50% of the farm households, who reported to be using such information for making their farming decision including planting, harvesting, threshing times, irrigation decision, choice of varieties, use of pesticides and agrochemicals, and drying. Farm households also receive WCIS from their social circles and friends, suggesting that farmers in Pakistan receive WCIS from multiple sources.

## Methodology

### Sampling and data collection

**Figure 1:**
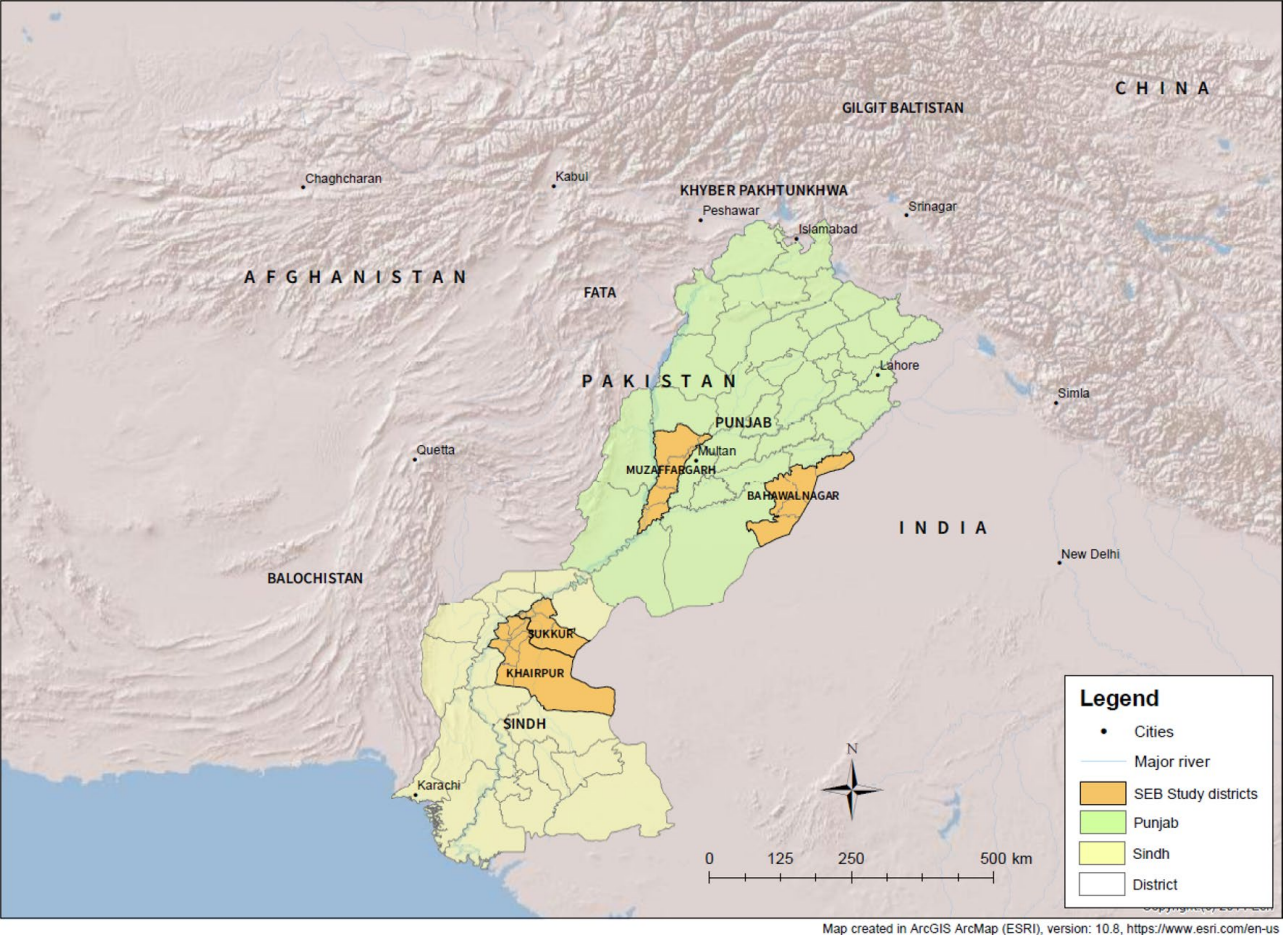
Location of study area. Source: The Authors.

To understand the impact of WCIS on farm level outcomes in Pakistan, we collected data from two provinces—Punjab and Sindh—where the cotton-wheat farming system is prevalent along with other crops. In this farming system, cotton and wheat are planted sequentially in a rotating system. In order to make our survey representative of the cotton-wheat cropping region in Pakistan, we used a multi-stage stratified cluster random sample technique^30^. The two provinces—Punjab and Sindh—were selected due to their vulnerability to floods and droughts and the predominance of cotton-wheat farming system. This involved stratifying the two provinces by randomly selecting 4 districts from the stratified areas, two from each province. From each district, we selected two Tehsils randomly (total 8 Tehsils), and from each Tehsil, two Union Councils from Punjab and one Union Council from each Tehsil from Sindh (as Sindh has relatively smaller cotton-wheat cropping area) were selected, making a total of 12 Union Councils in the sample. Then from each Union Council, we randomly selected two villages (a total of 24 villages), and from each village, we randomly selected 2 wards (the lowest level of the administrative unit with the exception that we selected three wards from two of the villages) making a total of 50 wards in our sample. Then from each ward, we randomly selected 10–15 households for the survey (612 households in total).We surveyed women members of the household from every alternative household in the sample, making our survey gender balanced (49.2% of respondents were women). Figure 1 highlights the study area, and Table S1 (supplementary materials) shows the sampled villages and related information.

We conducted the farm households survey in two phases, in order to collect information for the two crops—cotton and wheat. The same farm households were visited by the enumerators in both phases of the survey. In the first phase, we collected farm household’s socio-economic and demographic information along with information related to wheat farming as this coincided with the wheat harvesting season of 2021 (April). The second phase of survey was conducted during the cotton harvesting season of 2021 (October) and focused solely on collecting information related to cotton production. We followed relevant guidelines and regulations while collecting data and information.

Focus group discussions (FGDs) were also conducted during the second round of the farm household survey to help explore in depth the use of WCIS by farmers. Using the same villages surveyed in the second round, 64 FGDs were pursued with users of WCIS in the two provinces. Similarly, to the survey, these FGDs were also organised by gender in order to facilitate discussions. A total of 36 FGDs with male participants and 28 FGDs with female participants were conducted. The FGDs explored a number of topics including the benefits of using WCIS, the barriers farmers encountered when using this information, and how it could be enhanced to further facilitate its use in their decision-making processes.

The enumerators responsible for implementing the survey and FGDs on the ground were trained by the research team to ensure consistency on how questions were asked, data was recorded as well as how to approach respondents and organize the FGDs. In addition, 50% of the enumerators were women and responsible for conducting the survey and FGDs with female respondents to help manage local culture and expectations in relation to gender issues.

### Basic model

**Figure 2:**
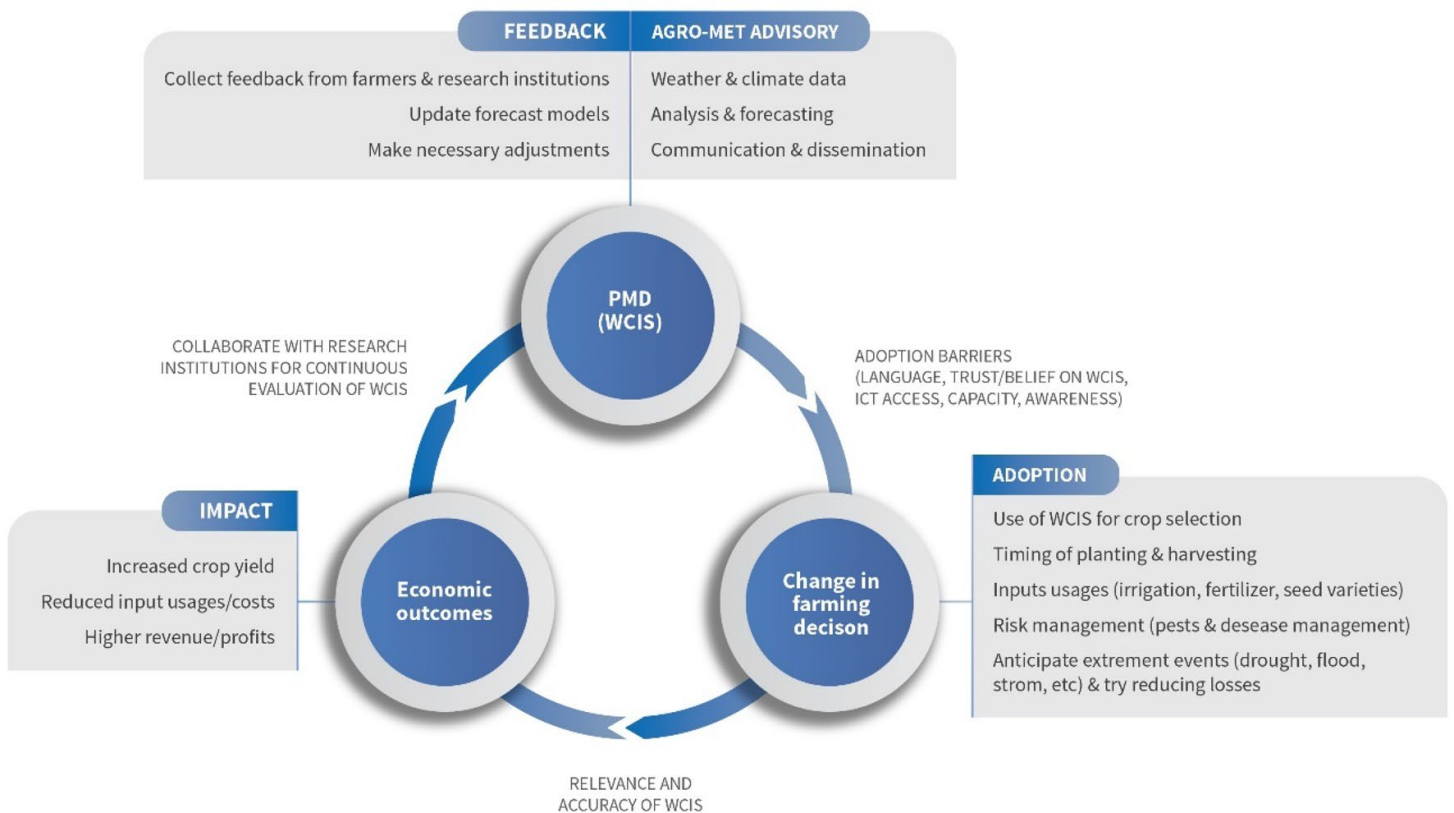
Framework for the link between WCIS and farm level outcomes. Source: The Authors.

Since our objective is to understand the impact of using WCIS, on farm outcomes (cost, revenue, profits, and inputs usage), we posit our theoretical framework where information is treated as an aid to the farming decisions that help save input uses or enhance productivity e.g. with the right combination of inputs or choosing right timing for establishing crops, applying irrigation water, fertilizer, and agro-chemicals as needed for reducing the risk of crop loss, and harvesting on time. Figure 2 provides a framework of our study, where assumptions regarding the collection, dissemination and uptake of the WCIS are clearly outlined.

Information economics provides a framework for the valuation of information used for making decisions in a variety of cases^31^. One of the most useful areas for its application is on the use of WCIS in agriculture. The approach of valuing WCIS begins with the agent using the information as an input in their farming-related decision-making to reduce uncertainty^32^.

Johnson and Holt^32^ assume that an agent needs to choose from a finite set of actions,* a*, while performing agricultural activities under the states of nature (weather or climatic events), *s*, that are external to the agent, which will create combinations, *c(a, s)*, of actions and states. The utility function of the agent is a function denoting all possible combinations of actions and states, *u[c(a,s)]*. Uncertainty is prevalent as the agent chooses an action without knowing the future state. The agent uses historical weather information pertaining to their subjective probability, *p*. Bayesian approach is used to determine the probabilities of different states of the world given that weather information received by the agents changes their subjective probabilities. Then, the agents maximize their expected utility by selecting an optimal action.

The relationship between likelihood function, posterior probability, and prior probability relating to receiving the climate information and states of the world provides agents with choices to act upon before and after getting the information^32^. This association is often referred to as ‘hard’ and ‘soft’ prior beliefs about possible states of the world which in turn suggests a diffuse observation that those having great confidence (hard belief) in a particular state would not be likely to refer to the information provided to them.

So, in an ex-post position, the value of information is generally the difference between the expected utilities,

1 $${V}_{i}={E}_{i}\left\{u\left[c\left({a}_{i},.\right)\right]\right\}-{E}_{0}\left\{u\left[c\left({a}_{0},.\right)\right]\right\}$$

where*, V*_*i*_ is the net value of receiving WCIS message *i*, *i* = *1,…,I*, and *E*_*i*_*{u[c(a,.)]}* is the expected utility to the agent if action *a* is taken.

The agents must compare two choices, *a*_*0*_ and *a*_*i*_. Under *a*_*0*_, the agents choose an optimal action where the information has yet to be received (or used for making any decision) and is acting on historical information. Under *a*_*i*_, the agents choose an optimal action using the WCIS relayed by the message, *i*, in accordance with its posterior probability. The difference in the expected utilities of these two choices is the additional value of the WCIS received by the agents in an ex-post situation where the information has been known.

In an ex-ante situation, the agents will not know what kind of information they are going to get. Suppose the agents get the weather information from a public agency, such as the Pakistan Meteorology Department, which is free of cost. This provides a new decision problem where they must factor in the probability of receiving the information of which the agents have no knowledge or whether the agents should use the information for making their decision,

2 $${V}^{*}={\sum }_{i}{q}_{i}{E}_{i}\{u\left[c\left({a}_{i},.\right)\right]\}-{E}_{0}\left\{u\left[c\left({a}_{0},.\right)\right]\right\}$$

where,* q*_*i*_ is the joint probability of receiving message *i*, and using it for making their farming decision.

The agents need to integrate all the messages received and their respective probabilities to find the expected utility gain of using the information in the ex-ante valuation. This is a utility-based measure of value but there are ways to convert it into cash value depending on the nature of the risk taken by the agents^33^.

Using a similar framework, Meza et al.^33^ have devised a net expected value of forecast information (NEVOI) incorporating certain agricultural and meteorological inputs,

3 $$NEVOI=E\left\{U\left[Y\left(X\ddagger \right),{W}_{0}\right]\right\}-E\left\{U\left[Y\left({X}^{*}\right),{W}_{0}\right]\right\}$$

where the first expression on the right-hand side represents the expected utility using the weather forecast received $(X\ddagger )$ from the concerned agency, while the second expression is the expected utility using agents’ perception based on prior information (X*) but not the WCIS, and W refers to the wealth of the agent.

Other ways of valuing weather information include Von Neumann–Morgenstern utility framework and rational expectations theory. For example, Babcock^34^ used this approach to study whether improved weather forecasts contribute to improving farmers’ returns. Another approach could be the use of an optimization process, where one can use ex-post profit maximization or cost minimization framework. Based on these models, we use an econometric approach for evaluating the impact of the WCIS.

### Econometric methods and issues

In order to operationalize the theory into empirics, we estimated a reduced form econometric model. We developed an econometric model based on the premise that the outcomes from agriculture, *Y*_*ijk*_, depend on the use of WCIS, *CS*_*ijk*_, characteristics of the respondents, *CR*_*ijk*_, household characteristics, *HC*_*ijk*_, community characteristics including distance to input and output markets from the village, *CC*_*ijk*_, agricultural practices that the households choose (such as machine vs. manual harvesting), *AP*_*ijk*_, information sources that the households receive related to agricultural activities, *IS*_*ijk*_, and soil quality of the farmland, *FQ*_*ijk*_, that the household cultivates. Here, *i* refers to a household, *j* refers to the community (ward), and *k* refers to the districts that the household lives in. All these variables are vectors. For example, *Y*_*ijk*_, refers to farm households’ revenue, farming cost, and profit from either wheat or cotton crop or inputs used. On this backdrop, we estimated the following regression model:

4 $$Y_{ijk} = \, \beta_{0} + \, \beta 0_{1} CS_{ijk} + \, \beta_{2} CR_{ijk} + \, \beta_{3} HC_{ijk} + \, \beta_{4} CC_{ijk} + \, \beta_{5} AP_{ijk} + \, \beta_{6} IS_{ijk} + \, \beta_{7} FQ_{ijk} + \, d_{k} + \, \mu_{ijk}$$

where *d*_*k*_ denotes district level fixed effects to control for unobserved heterogeneity that may vary across the sampled districts, and *µ*_*ijk*_ refers to a white noise error term with zero mean and constant variance. These variables are summarised in Table 1, where the mean difference between WCIS user and non-user farm households is compared. This table also includes input usage for farming such as fertilizer, agrochemicals, and irrigation, which are the additional outcome variables we consider in this study.

**Table 1:** Descriptive statistics (mean difference) of the key outcome variables.

	Wheat			Cotton		
	Non-users	Users	Mean diff	Non-users	Users	Mean diff
Revenue	85.46	89.079	− 3.619***	125.734	120.622	5.112**
Cost	41.681	45.475	− 3.794***	111.699	152.69	− 40.991***
Profit	43.779	43.604	0.174	14.035	− 32.069	46.104***
Fertiliser Cost	13.215	12.821	0.394	31.682	30.399	1.282
Chemicals Cost	3.214	3.379	− 0.165	34.43	63.256	− 28.826***
Irrigation Cost	3.733	6.773	− 3.041***	15.049	29.491	− 14.442***

n = 612, n_1_ = 272 (non-users of WCIS) and n_2_ = 340 (users of WCIS); ** and *** indicate a significant difference between non-users and users of WCIS at 5% and 1% level; all values are in PKR ‘000 per acre.

### Identification issue and approaches

Since the use of WCIS is not randomly assigned to farm households but households decide whether to access and use it, there may be selection bias in the estimated results using the ordinary least squared (OLS) method. Therefore, other than the OLS method, we use two alternative methods as the identification strategies: (a) instrumental variable approach, and (b) matching method.

#### Instrumental variable approach

Instrumental variable approach is one of the frequently used methods for addressing selection bias and resulting endogeneity issues in an observational study^35–38,40^. In our case, WCIS is public information provided by PMD or other sources, but farm households needed to have electronic devices such as mobile phones, televisions, and the Internet, to access the information. However, the WCIS that households could access may or may not be used in making farming decisions depending on their perception of the degree of climate risk the households might be facing, whether they can understand the WCIS coming from external sources (such as the use of a language other than their mother tongue) and whether the farmers had any prior experience of false alarms from WCIS in the past (Fig. 2). Since access to TV (86%) and mobile phone (93%) are prevalent among the sampled farm households (almost all farm households had both and hence low variation), we used different variables as instruments including: whether the farmers understand the WCIS well, whether they have experience of false alarms in the past (due to less accurate WCIS), and if the households have access to the Internet. We believe that these variables affect whether the farm household *uses* the WCIS, but there is no direct link between these variables and the farm outcomes (profit, revenue, cost, and input usage), justifying the use of these variables as the instrument. We provide relevant statistics to support this claim in the results section.

#### Propensity score matching

As an alternative to the OLS and IV approaches, we also used the propensity score matching approach for estimating the impact of WCIS while using observational data^41–46^. In this approach, farm households are matched based on their observed characteristics, and farm-level outcomes are compared between two comparable groups of households based on their observed characteristics with the only difference being that one group uses WCIS and the other does not. In this case, the assumption is that any difference observed can be attributed to the use of WCIS as other similar observable characteristics are common to both groups. While estimating the propensity scores, we use all variables that are used as control variables for OLS or IV estimation. Since this approach mimics the experimental approach for reducing the selection bias based on the observed characteristics, it is often referred to as a quasi-experimental approach for estimating the causal impact of the variable in question^47,48^.

Another option could be to estimate the production function and compare the marginal productivity of the inputs used by the WCIS user households in comparison to the non-user households. However, in the farming system, inputs are generally used proportionately per unit of land and hence one can face a high degree of multicollinearity among the inputs^49^. Therefore, we did not estimate the production function to analyze the impact of WCIS use on farm outcomes.

### Ethics approval and consent to participate

SANDEE Secretariat reviewed the survey design and instruments to ensure that it met the ethical standards and protect the privacy and voluntary participation of the respondents. Verbal informed consent was obtained before engaging with the respondents.

### Consent for publication

ICIMOD’s Publishing and Outreach Committee (POUT) reviewed and approved the manuscript before submitting it to the journal. All authors agree to publish the joint work.

## Results

### Descriptive statistics

Table 1 indicates a significant difference in the means of the farm-level outcomes considered in this study except for the cost of fertilizer, and chemicals cost (for wheat crops) between users and non-users of the WCIS. However, as our study is based on one round of observational data, we cannot conclude anything based on the mean differences alone.

In Table 2, we present the characteristics of the farm households, other controls, and the instruments used for the study.

**Table 2:** Mean difference of the variables used for the analysis.

Variables	WCIS non-users	WCIS users	Mean Diff
Respondent characteristics			
Female respondent (1/0)	0.522	0.468	0.054
HH head as the respondent (1/0)	0.489	0.432	0.057
Highest education of household member			
Below high school (1/0)	0.191	0.171	0.021
High school (1/0)	0.257	0.288	− 0.031
Intermediate level (1/0)	0.188	0.188	− 0.001
Graduate level (1/0)	0.173	0.291	− 0.118***
Other characteristics of the household/community			
Distance to market (km)	8.636	9.443	− 0.807
Received agri training (1/0)	0.221	0.506	− 0.285***
Income source—govt service (1/0)	0.107	0.126	− 0.02
Received extension service (1/0)	0.21	0.494	− 0.285***
Operational land holding (ha)	6.555	11.175	− 4.620***
Machine harvesting (1/0)	0.228	0.356	− 0.128***
No of land parcels	2.004	1.894	0.11
Cooking with LPG (1/0)	0.184	0.082	0.101***
Drought occurred in the past 10 years (1/0)	0.401	0.391	0.01
Received warning for natural disasters (1/0)	0.32	0.812	− 0.492***
Fertile land (1/0)	0.596	0.488	0.107***
Instruments			
Understand WICS (1/0)	0.004	0.559	− 0.555***
Perceives WICS may provide false alarm (1/0)	0.004	0.532	− 0.529***
Access to internet (1/0)	0.143	0.353	− 0.210***
No of observations	272	340	

n = 612, n_1_ = 272 (non-users of WCIS) and n_2_ = 340 (users of WCIS); ** and *** indicate significant difference between non-users and users of WCIS at 5% and 1% level.

From Table 2, we can infer that the farm households’ characteristics are similar in terms of demographic, education, distance to market, income source, number of land parcels, and occurrence of drought in the past 10 years. Other variables, such as receiving extension services and agriculture-related training, receiving warnings for natural disasters, land holding size, and use of machines for harvesting, are systematically different in two groups with the higher mean value of these variables for the users of WICS, suggesting that the users can potentially be relatively large landholders, receiving more extension services, and getting more agriculture-related training.

## Econometric results

### Impact of WCIS on revenue, cost and profit

As we have controlled for a large number of variables and estimated results separately for each crop, we report the key estimates in Table 3 by crop type for the three key outcomes: profit, revenue and cost (per acre), where Supplementary Materials (Tables S2 and S3) provide detailed results for both crops. Since the farmers could incur negative profit, we used profits in thousand PKR (Pakistani Rupee) and used log transformation for revenue and cost per acre to address the scale issues. In Table 3, we summarize the results from all three methods (OLS, IV, and PSM) used for estimating the impact of WCIS.

**Table 3:** Effect of WCIS on agricultural revenue, cost and profits (per acre) for wheat and cotton crops.

Estimation methods	Profit ('000)		Revenue (log)		Cost (log)	
	Wheat	Cotton	Wheat	Cotton	Wheat	Cotton
OLS	− 1.02	− 11.26	0.01	− 0.01	0.04	0.06
(SE)	(1.55)	(7.80)	(0.01)	(0.03)	(0.02)	(0.05)
R-squared	0.12	0.38	0.26	0.24	0.41	0.51
IV-Estimates	− 1.24	− 2.84	0.01	− 0.00	0.02	0.03
(SE)	(2.92)	(14.29)	(0.02)	(0.05)	(0.04)	(0.08)
PS Matching (PKR, level)	− 1489	− 7370	1218	− 4291	2707	3089
(SD)	(1848)	(9816)	(1522)	(4390)	(1398)	(9503)
Sample mean and SD (PKR '000)	43 (15)	(-12) (85)	87(12)	123(29)	44(12)	134(84)
Sample Size	612	612	612	612	612	612

We controlled for all variables listed in Table 2 (except the instruments), and also used district fixed effects to account for the unobservable differences across the districts. For OLS and IV approaches, profit is measured in PKR ‘000, and revenue and cost are log-transformed. For PS matching, all three outcomes are in level (PKR); clusters (ward level) robust standard errors are in parentheses; ***p < 0.01, **p < 0.05, *p < 0.1

For IV estimates, we used three variables as instruments (as discussed in section "Instrumental variable approach" and also listed in Table 2 as *instruments*). The first stage F-statistics is 28.4 which is much higher than 10, suggesting that the instruments that we used are reasonably strong. The Anderson canonical correlation likelihood ratio statistics is 278 (*p* < 0.001), suggesting that the model is identified. The IV estimates are also robust to the change in the IVs (when we dropped access to the internet as one of the IVs, the first stage F-stat slightly increased to 35.9).

**Figure 3:**
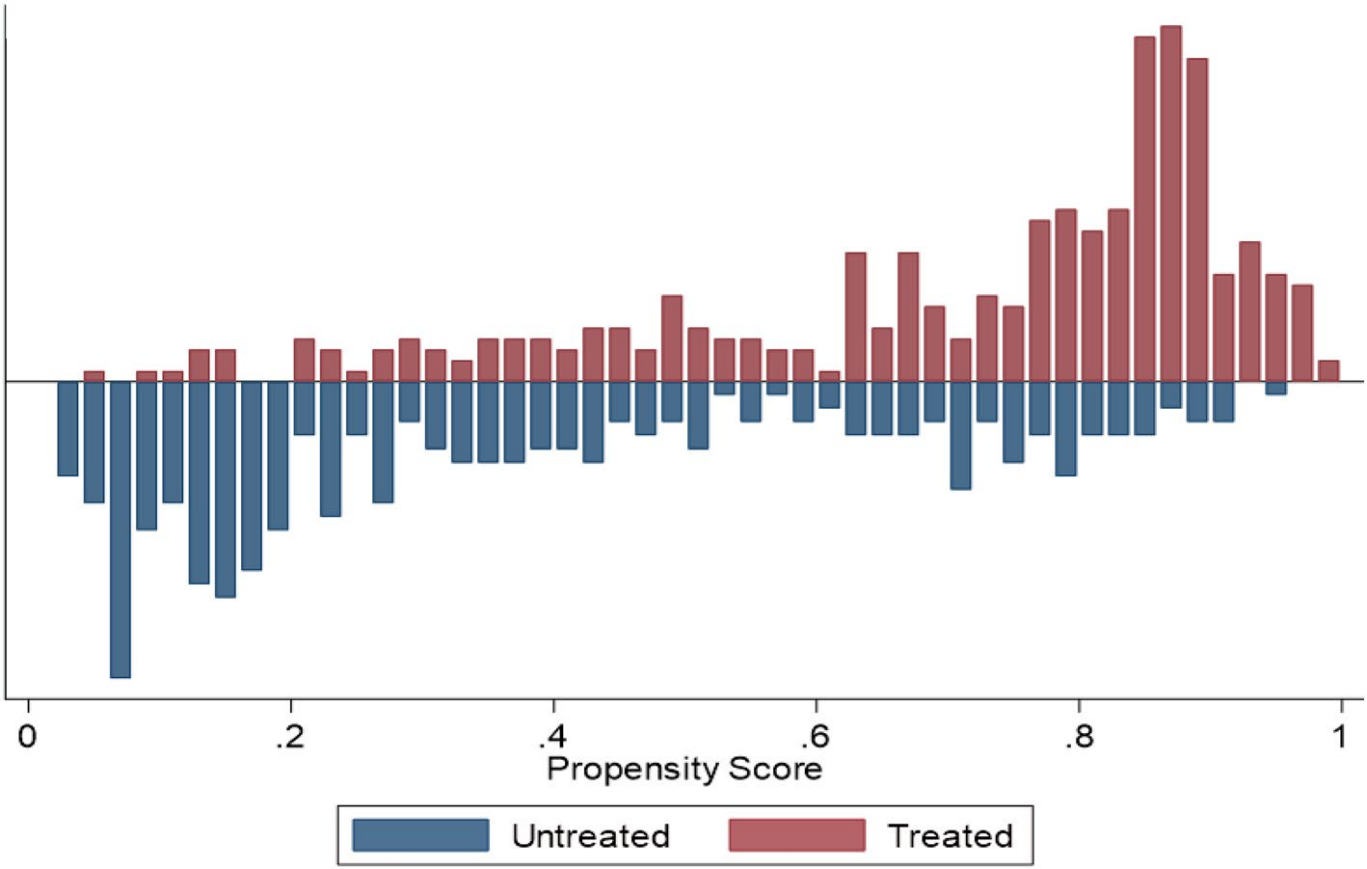
Common support and estimated propensity scores. Note: In this context, untreated refers to non-users and treated refers to the users of WCIS. In some cases, there is no common support or overlaps between households who were users and non-users of the WCIS who have the same propensity scores. Such unsupported observations were dropped for PSM analysis.

The propensity score matching approach suggests that there is a significant overlap or common support between the farm households who used WCIS and those who did not, based on their observable socioeconomic and other characteristics. Figure 3 displays the common support. For PSM analysis, we dropped the farm households that did not have common support in order to generate comparable groups of users and non-users of WCIS so that the impact of the WCIS can be estimated.

**Figure 4:**
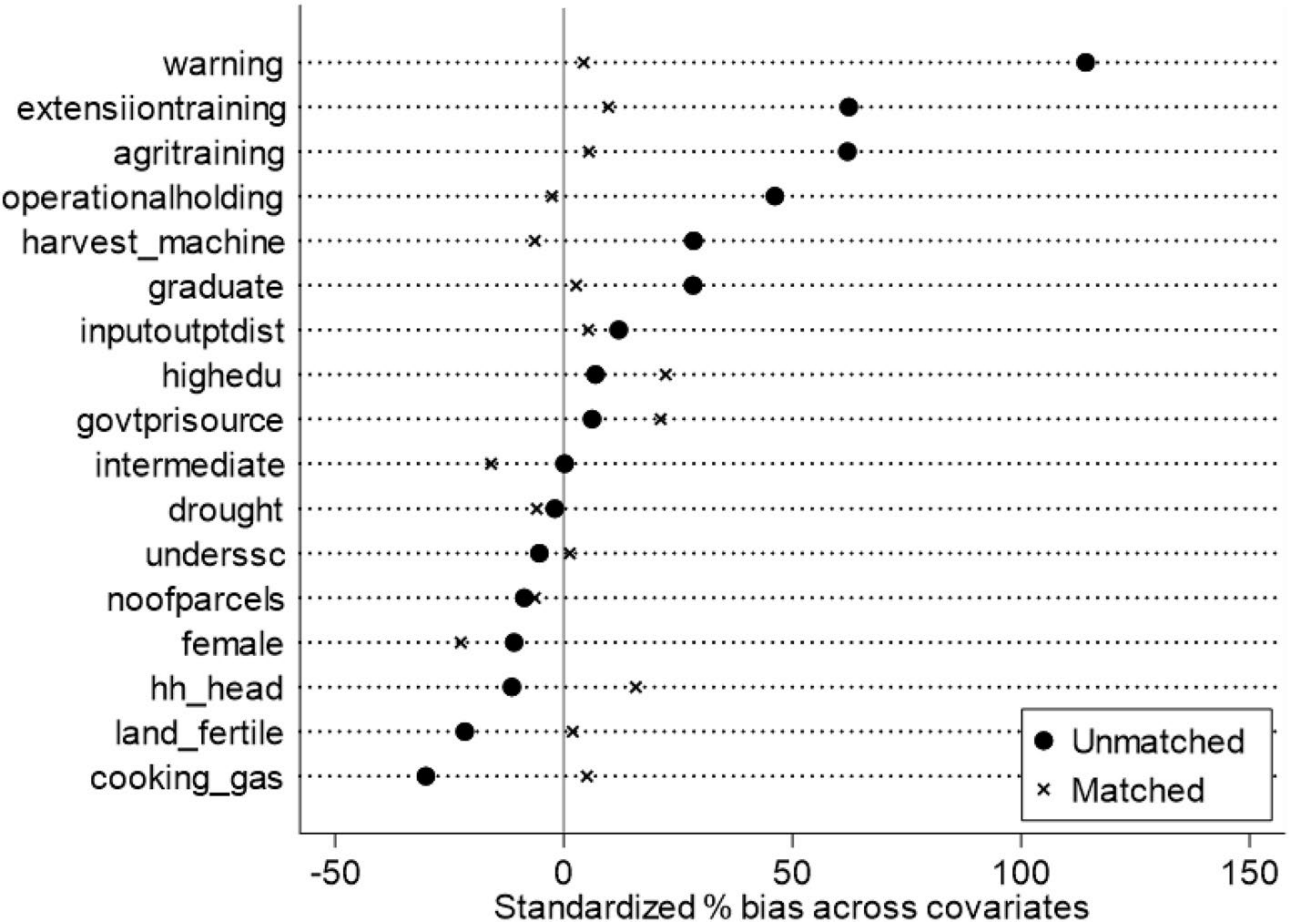
Reduction in standarized bias with propensity matching.

We have also analyzed the quality of matching based on the comparison between the standardized biased before and after matching for each covariate. Overall, Fig. 4 suggests that the standardized bias has been reduced significantly with matching, from over 29% pre-matching to 9% post-matching on average.

In Table 3, we provide the key results from all three different estimation methods where the outcome variables are per acre revenue, cost, and profits for both crops (wheat and cotton). Since we control for several characteristics of the farm households, we just report the coefficient of the main variable of interest, *users* (it indicates whether the households use WCIS), in this table. We provide the detailed results for the IV estimates in Table S2 (supplementary materials). Table 3 shows that none of the estimated coefficients of the variable *users* is statistically significant, suggesting that farmers who have been using WCIS do not seem to benefit in terms of farm revenue, cultivation cost reduction, and/or profits. This result is consistent for all three different approaches that we have used for estimating the impact of using WCIS for both crops.

### Impact on agricultural inputs

Farmers may change their agricultural practices depending on weather and climate forecasts. For example, if rain is forecasted for the next 24 h, they may wait rather than irrigating their crop the following day. This ability to decide based on WCIS can help save water, and related costs for the farmer. In our analysis, we consider the impact of WCIS on the cost of using three key inputs that have environmental implications—irrigation, chemical fertilizer, and agrochemicals. Table 4 shows the estimated impact of the WCIS use on the cost of three key inputs, where we report the coefficient of the key variable of interest, *users of WCIS*. Table S3 (supplementary materials) shows the detailed results from the IV estimates. Since the OLS estimate suffers selection bias, we did not report the detailed results of OLS estimates in the supplementary materials.

**Table 4:** Effect of WCIS on cost of irrigation, fertilizer, and agro-chemicals usage.

Estimation methods	Cost of Fertiliser (log)		Cost of Chemicals (log)		Cost of Irrigation (log)	
	Wheat	Cotton	Wheat	Cotton	Wheat	Cotton
OLS-Estimates	0.02	− 0.01	0.03	0.14*	0.08	− 0.29
(SE)	(0.03)	(0.08)	(0.09)	(0.08)	(0.09)	(0.18)
R-squared	0.25	0.26	0.23	0.64	0.62	0.28
IV-Estimates	− 1.11	− 2.92	0.01	− 0.00	0.02	0.04
(SE)	(2.81)	(14.19)	(0.02)	(0.05)	(0.04)	(0.08)
PM Matching (level)	− 67	− 445	221	2278	1201*	1671
(SD)	953	3738	253	6219	628	3940
Sample Mean &SD (PRK '000)	13(8)	31(30)	3(2)	50 (54)	5 (6)	23 (39)
Sample Size	612	612	612	612	612	612

We controlled for all variables listed in Table 2 (except the instruments), and also used district fixed effects to account for the unobservable differences across the districts. All outcome variables are log-transformed. For PS matching, all three outcomes are in level (PKR); clusters (ward level) robust standard errors are in parentheses; ***p < 0.01, **p < 0.05, *p < 0.1

Our results displayed in Table 4 suggest that the expenditure on fertilizer is not affected by the use of WCIS for cotton and wheat crops. In both IV and matching models, the expense on fertilizer seems to be reduced to a certain extent (a negative sign of the estimates) however this reduction is not statistically significant. For the expenses on chemicals, OLS results for cotton show some increment, but this evidence does not sustain in IV and matching models. There is also very weak evidence (significant at 10% level) that the expense on irrigation is a bit higher (PKR 1201/acre) for the WCIS users in comparison to non-users for wheat crop when we use the matching method, which is around 24% of the sample mean. Since the standard error is quite wide, there is no strong evidence of the increased expenses on irrigation for the farmers who used WCIS.

### Focus group discussion

Additional qualitative information was collected through the FGDs to help us better understand why farmers were not getting the expected economic benefits from WCIS they use. A key finding from the FGDs conducted was that, although around 55% of the farm households surveyed claimed to be using WCIS, many also indicated that they do not trust the reliability of the information provided. This was either due to the information not being perceived as accurate or being too general (in terms of spatial scale) for farmers to be able to apply the information to their specific decisions. The timeliness of the information (in order for farmers to be able to act on time) and accessibility issues e.g. power cuts or mobile network charges were also identified as barriers for farmers to use WCIS. Farmers agreed that removing these barriers would allow them to make better use of available WCIS. Additional improvements included tailoring WCIS to better fit farmers’ needs e.g. information based on specific crops, further training and support to educate farmers on how to act upon under unexpected weather conditions, better use of WCIS, and better support on the ground from experts such as extension officers.

## Discussion

In our sample, over 55% of the farm households who cultivated wheat and cotton were using WCIS in 2021. Despite this, our analysis shows a lack of evidence on the effectiveness and impact of using WCIS on key farm-level outcomes such as increased agriculture revenue and profits, reduced costs, and input usage including irrigation, fertilizer, and use of agrochemicals for reducing crop loss risks and increasing crop yield. Our results are consistent with what Sharma et al.^26^ found in India where farmers did not change the applications of fertilizer and agrochemicals after receiving WCIS in an experimental setting. A study from Zimbabwe^50^ reports similar results where the use of WCIS did not improve the yield of pearl-millet. These findings also align with Babcock^34^, where the author demonstrates that improved weather forecasts does not necessarily benefit the farmers who face inelastic (less responsive) demand with respect to price change.

Since we use observational data with a seasonal recall of 4–5 months, there have been some possibilities of recall bias for the accuracy of the reported information on farm-level outcomes (revenue, cost, and profit), and input usages^51^. However, since we have collected information from farm households who are both the users and non-users of WCIS, we expect that both groups have comparable recall bias and hence the results that we report are less biased one way or the other. Furthermore, as we collected information right around harvesting time, farmers are expected to remember what inputs they have used, how much they have spent, and quantity of each crop that they have harvested, leading to a minimal level of recall bias.

The lack of evidence between the use of WCIS and such impact on major farm outcomes from the statistical analysis pursued in this study can be explained, at least to a certain extent, by a number of aspects that were identified and discussed during the FGDs with the farmers.

For example, whilst survey findings showed that 55% of the farm households currently use WCIS to support a range of agricultural-related decisions, much of the WCIS used (i.e. daily weather forecasts and farmers advisories) are received by farmers via National TV or SMS. This potentially indicates a more diluted use of WCIS by farmers in that, accessing this information does not necessarily translate into its direct application in specific decisions^52^. This is further substantiated by the FGDs where farmers described the range of barriers and difficulties they face when trying to use WCIS.

In addition, in poor and marginalized contexts where resources can be limited and the risk attached to specific decisions can have significant impacts on livelihoods, the use of WCIS can end up being more of a consideration when making decisions rather than a direct input to that decision-making process^52^^,^^53^. Trust in the source of WCIS can also influence the use of WCIS particularly when the information provided is more uncertain (e.g. information with longer lead time such as monthly and seasonal forecasts)^54^.

In addition, participants who filled in the survey and participated in FGDs may access and use WCIS but they may not necessarily have the agency to make agricultural-related decisions in the household^53^ particularly when considering gender aspects. These various factors could help explain the lack of significant differences in economic benefits from users and non-users of WCIS. Our study suggests that quantitative research should be supplemented by qualitative information (e.g. FGDs) in order to understand why there is effect or no effect of any intervention and what can be done to improve the situation on the ground.

## Conclusion

Weather and climate events have been rapidly growing globally due to changing climate conditions leading to unprecedent impacts and consequences to farming communities^1^. In this context, the use of WCIS can play a key role in supporting farmers to better prepare for such impacts. However, the literature on the link between the use of WCIS and farm level outcomes is currently limited. This study aims to fill this gap by addressing the following research question: can the availability and use of climate information services (WCIS) help improve farm level outcomes given the uncertainty around weather and climate variability? To understand the impact of WCIS in cotton-wheat cropping areas of Pakistan, we conducted a multistage stratified clustered random sample of 612 farm households from two provinces. We analyzed the survey data using different model specifications (liner as well as non-liner) and estimation techniques (OLS, IV and PSM). Over 55% of the farm households in the sample indicated that they used WCIS provided by the Pakistan Meteorological Department and other sources for making their agricultural decisions. Our analysis, however, suggests that the impact of using WCIS on major farm outcomes (farm revenue, costs, profits, and inputs usage) is not statistically different from the farm households who do not use such information (null result).

Since Pakistan is one of the most vulnerable countries in South Asia to climate change due to frequent and extreme climate events, key livelihoods in the country such as agriculture dependent farming community should be able and capacitated to benefit from the use of WCIS to help them effectively adapt to climate variability and change. For this to happen, responsible authorities such as PMD should consider the above-mentioned constraints faced by farmers to help improve WCIS and increase its use and economically benefit these communities. WCIS can be improved through research, training, and continuous evaluations to support farmers in gaining trust, learning to use it and increase its uptake to achieve intended benefits of WCIS. As most of the Least Developed Countries in South Asia and elsewhere face similar challenges, our findings and recommendations can be applied in similar contexts.

### Supplementary Information

Supplementary Information.

## Data Availability

Data will be available from the corresponding author upon reasonable request.
