# Supplementary material for: Impact of weather and climate advisories on agricultural outcomes in Pakistan
Source: Sci Rep. 2024 Jan 10;14:1036. doi: 10.1038/s41598-023-51066-4 (PMC10781742; doi:10.1038/s41598-023-51066-4)
Supplement: Supplementary file 1 — Supplementary Information. [file 41598_2023_51066_MOESM1_ESM.docx]

**Supplementary Materials**

**Impact of weather and climate advisories on agricultural outcomes in Pakistan**

Mani Nepal^1, 2^*, Muhammad Ashfaq^3^, Bibek Raj Sharma^1,2^, Mandira Singh Shrestha^2^, Vijay Ratan Khadgi^2^ and Marta Bruno Soares^4,5^

**—-------------------------------------------**

^1^ South Asian Network for Development and Environmental Economics (SANDEE), Nepal

^2^ International Center for Integrated Mountain Development (ICIMOD), Nepal

^3^ Muhamad Nawaz Shareef University of Agriculture, Multan, Pakistan

^4^ University of Leeds, United Kingdom

^5^ Met Office, United Kingdom

* The corresponding author, mailing address: GPO Box 3226, Kathmandu, Nepal. T +977-1-5275222, Ext: 412; email: [mani.nepal@icimod.org](mailto:mani.nepal@icimod.org); [mani.nepal@bus.illinois.edu](mailto:mani.nepal@bus.illinois.edu).

Table S1: Number of respondents per Province, District, Tehsil, UC and Village

| Province | District | Tehsil | Union Council | Village |
| --- | --- | --- | --- | --- |
| Punjab  (N= 413) | Bahawalnagar  (N= 211) | Haroonabad  (N= 110) | 42/3R  (N= 48) | 36/3R (N= 26) |
|  |  |  |  | 42/3R (N=22) |
|  |  |  | 432/6R  (N= 62) | 428/6R (N= 32) |
|  |  |  |  | 432/6R (N= 30) |
|  |  | Chishtian  (N= 101) | 169 Murad  (N= 47) | 170/M (N= 24) |
|  |  |  |  | 138/M (N= 23) |
|  |  |  | 128 Murad  (N= 54) | 129/M (N= 30) |
|  |  |  |  | 126/M (N= 24) |
|  | Muzafargarh  (N= 202) | Kot Addu  (N= 96) | UC22, Budh  (N= 45) | Chakien wala (N= 23) |
|  |  |  |  | Marhien wala (N= 22) |
|  |  |  | UC-10, Dogar Kalasara  (N= 51) | Khar Gharbi (N=22) |
|  |  |  |  | Daya Chowk Gharbi (N= 29) |
|  |  | Ali Pur  (N= 106) | Dummer Wali  (N= 48) | Basti Gabol (N= 24) |
|  |  |  |  | Makhan Bela (N=24 ) |
|  |  |  | Ghalwan  (N= 58) | Ghalwan Shehr (N=23) |
|  |  |  |  | Tibbi Arain (N= 35) |
| Sindh  (N= 199) | Sukkur  (N= 100) | Saleh Patt  (N= 51) | Saleh Patt  (N= 51) | Saleh Putt (N= 27) |
|  |  |  |  | RD-71 (N= 24) |
|  |  | Pano Aqil  (N= 49) | Dadloi  (N= 49) | Sahib Khan Korai (N= 26) |
|  |  |  |  | Nubaho Malik village (N= 23) |
|  | Khair Pur  (N= 99) | Kingri  (N= 50) | Kulaib Jail  (N= 50) | Kolab Jial (N= 25) |
|  |  |  |  | Shakaruddin Pujabi (N=25) |
|  |  | Kot Diji  (N= 49) | Talpur Wada  (N= 49) | Abdul Raheem Kaskheli  (N= 25) |
|  |  |  |  | Babad Wada (N= 24) |
| Total | 4 | 8 | 12 | 24 |

Table S2: Detailed results from IV Approach for Profit, Revenue and Cost

|  | (1) | (2) | (3) | (4) | (5) | (6) | |
| --- | --- | --- | --- | --- | --- | --- | --- |
|  | Profit ('000 PKR) | | Revenue (log) | | Cost (log) | | |
| VARIABLES | Wheat | Cotton | Wheat | Cotton | Wheat | Cotton | |
| Users of WCIS | -1.24 | -2.84 | 0.01 | -0.00 | 0.02 | 0.03 | |
|  | (2.92) | (14.29) | (0.02) | (0.05) | (0.04) | (0.08) | |
| Female respondent | -1.32 | 50.87*** | -0.01 | 0.11*** | 0.02 | -0.22*** | |
|  | (2.59) | (11.20) | (0.02) | (0.02) | (0.04) | (0.07) | |
| Distance to input/output market | 0.22* | 2.10*** | 0.00 | 0.00 | -0.00* | -0.01*** | |
|  | (0.12) | (0.57) | (0.00) | (0.00) | (0.00) | (0.00) | |
| HH Head | -1.98 | 2.24 | -0.02 | -0.00 | -0.01 | -0.03 | |
|  | (2.10) | (10.38) | (0.02) | (0.03) | (0.03) | (0.06) | |
| Education – below high school | -1.39 | 12.21 | -0.06*** | -0.01 | -0.07** | -0.05 | |
|  | (2.51) | (10.85) | (0.02) | (0.03) | (0.03) | (0.06) | |
| Education – high school | -1.62 | 29.71*** | -0.03* | 0.01 | -0.02 | -0.16** | |
|  | (2.43) | (9.92) | (0.02) | (0.03) | (0.03) | (0.06) | |
| Education - intermediate | 2.12 | 15.45 | -0.01 | 0.02 | -0.06** | -0.10 | |
|  | (2.69) | (11.01) | (0.02) | (0.02) | (0.03) | (0.07) | |
| Education - graduate | 1.07 | 21.68** | -0.01 | 0.00 | -0.05 | -0.12** | |
|  | (2.66) | (9.04) | (0.02) | (0.03) | (0.03) | (0.06) | |
| Agri-training | -5.22 | 41.21** | 0.01 | 0.03 | 0.08 | -0.19 | |
|  | (5.48) | (18.41) | (0.04) | (0.06) | (0.09) | (0.12) | |
| Govt prisource | 2.10 | -14.71 | 0.02 | -0.02 | -0.00 | 0.08 | |
|  | (2.19) | (10.30) | (0.02) | (0.03) | (0.03) | (0.06) | |
| Extension training | 8.87 | -46.91** | 0.03 | 0.01 | -0.09 | 0.25** | |
|  | (5.51) | (19.14) | (0.04) | (0.06) | (0.09) | (0.13) | |
| Operational holding | 0.08 | 0.14 | 0.00*** | 0.00*** | 0.00** | 0.00 | |
|  | (0.08) | (0.35) | (0.00) | (0.00) | (0.00) | (0.00) | |
| Machine harvest | 1.80 | 13.15* | -0.02 | 0.06** | -0.10*** | -0.03 | |
|  | (1.34) | (7.73) | (0.01) | (0.03) | (0.03) | (0.05) | |
| No of parcels | 0.04 | -4.80* | 0.01** | 0.01 | 0.02 | 0.04** | |
|  | (0.99) | (2.58) | (0.01) | (0.01) | (0.02) | (0.02) | |
| Cooking gas | 1.03 | -5.22 | 0.00 | 0.01 | -0.03 | 0.07 | |
|  | (2.35) | (6.43) | (0.02) | (0.03) | (0.04) | (0.05) | |
| Drought | -1.59 | 3.48 | -0.06*** | -0.05** | -0.08** | -0.04 | |
|  | (1.71) | (9.13) | (0.02) | (0.02) | (0.03) | (0.05) | |
| Received disaster/weather warnings | 2.48 | 3.54 | 0.03** | -0.03 | 0.02 | -0.05 | |
|  | (1.77) | (10.83) | (0.01) | (0.04) | (0.02) | (0.05) | |
| Land fertile | 0.83 | -0.03 | 0.04*** | 0.03* | 0.05* | 0.04 | |
|  | (1.68) | (7.63) | (0.01) | (0.02) | (0.03) | (0.04) | |
| Constant | 34.56*** | -88.95*** | 11.34*** | 11.44*** | 10.82*** | 11.91*** | |
|  | (3.87) | (21.54) | (0.03) | (0.06) | (0.05) | (0.13) | |
| Observations | 612 | 612 | 612 | 612 | 612 | 612 | |
| R-squared | 0.12 | 0.38 | 0.26 | 0.24 | 0.41 | 0.51 | |
| Note: Robust standard errors in parentheses; *** p<0.01, ** p<0.05, * p<0.1; District fixed effects are used in all models. | | | | | | |  |

Table S3: Detailed results from IV Approach for Input Costs

|  | (1) | (2) | (3) | (4) | (5) | (6) |
| --- | --- | --- | --- | --- | --- | --- |
|  | Fertilizer Cost (log) | | Agro-Chemical Cost (log) | | Irrigation Cost (Log) | |
| VARIABLES | Wheat | Cotton | Wheat | Cotton | Wheat | Cotton |
| users | 0.07 | 0.04 | 0.11 | 0.07 | 0.15 | -0.48 |
|  | (0.06) | (0.13) | (0.10) | (0.14) | (0.15) | (0.30) |
| female | 0.05 | 0.05 | 0.14 | -0.33*** | 0.11 | -0.62*** |
|  | (0.06) | (0.11) | (0.09) | (0.11) | (0.12) | (0.19) |
| inputoutptdist | 0.00 | 0.01 | -0.01 | -0.02*** | -0.01 | -0.03** |
|  | (0.00) | (0.01) | (0.01) | (0.00) | (0.01) | (0.01) |
| hh_head | -0.04 | -0.01 | -0.08 | -0.09 | -0.08 | -0.37* |
|  | (0.05) | (0.08) | (0.12) | (0.08) | (0.08) | (0.20) |
| underssc | -0.07 | 0.01 | 0.09 | -0.10 | -0.07 | -0.27 |
|  | (0.05) | (0.12) | (0.15) | (0.11) | (0.16) | (0.24) |
| highedu | 0.04 | -0.14 | 0.13 | -0.24** | 0.04 | -0.20 |
|  | (0.06) | (0.10) | (0.18) | (0.10) | (0.20) | (0.21) |
| intermediate | -0.04 | -0.05 | 0.15 | -0.12 | -0.14 | -0.41* |
|  | (0.06) | (0.11) | (0.16) | (0.09) | (0.18) | (0.25) |
| graduate | -0.02 | -0.06 | 0.17 | -0.12 | -0.14 | -0.20 |
|  | (0.06) | (0.12) | (0.17) | (0.08) | (0.19) | (0.18) |
| agritraining | 0.35 | -0.51** | 0.29 | 0.13 | 0.04 | 0.10 |
|  | (0.23) | (0.24) | (0.23) | (0.10) | (0.16) | (0.48) |
| govtprisource | 0.05 | 0.12 | -0.12 | 0.16 | -0.04 | 0.01 |
|  | (0.05) | (0.09) | (0.16) | (0.10) | (0.08) | (0.24) |
| extensiiontraining | -0.32 | 0.78*** | -0.07 | -0.13 | -0.03 | 0.29 |
|  | (0.23) | (0.26) | (0.23) | (0.11) | (0.18) | (0.43) |
| operationalholding | 0.01*** | 0.01 | 0.00 | -0.00 | 0.01 | 0.00 |
|  | (0.00) | (0.00) | (0.00) | (0.00) | (0.00) | (0.01) |
| harvest_machine | 0.06 | -0.05 | -0.20** | -0.02 | -0.03 | 0.06 |
|  | (0.05) | (0.12) | (0.10) | (0.06) | (0.09) | (0.16) |
| noofparcels | 0.05* | 0.03 | 0.03 | 0.02 | -0.01 | 0.06 |
|  | (0.03) | (0.03) | (0.03) | (0.03) | (0.05) | (0.04) |
| cooking_gas | -0.01 | 0.07 | -0.05 | 0.11 | -0.30** | 0.08 |
|  | (0.09) | (0.08) | (0.19) | (0.07) | (0.14) | (0.18) |
| drought | -0.05 | 0.02 | -0.15 | 0.05 | -0.26** | -0.27* |
|  | (0.07) | (0.08) | (0.12) | (0.10) | (0.11) | (0.14) |
| warning | 0.03 | -0.06 | 0.02 | -0.12 | 0.01 | 0.32* |
|  | (0.04) | (0.08) | (0.09) | (0.08) | (0.09) | (0.19) |
| land_fertile | 0.05 | -0.09 | 0.13** | 0.10 | 0.07 | 0.13 |
|  | (0.05) | (0.08) | (0.06) | (0.06) | (0.08) | (0.20) |
| Constant | 8.84*** | 9.40*** | 7.85*** | 11.10*** | 9.19*** | 10.33*** |
|  | (0.10) | (0.13) | (0.21) | (0.18) | (0.23) | (0.49) |
| Observations | 612 | 612 | 612 | 612 | 612 | 612 |
| R-squared | 0.25 | 0.26 | 0.23 | 0.64 | 0.62 | 0.28 |
| Note: Robust standard errors in parentheses; *** p<0.01, ** p<0.05, * p<0.1; district fixed effects are used in all models. | | | | | | |
